# Seroprevalence of *Bordetella pertussis* specific Immunoglobulin G antibody levels among asymptomatic individuals aged 4 to 24 years: a descriptive cross sectional study from Sri Lanka

**DOI:** 10.1186/s12879-016-2068-z

**Published:** 2016-12-01

**Authors:** Shamithra Sigera, Jennifer Perera, Jeyanthakumar Rasarathinam, Dulani Samaranayake, Dileepa Ediriweera

**Affiliations:** 1 Department of Microbiology, Faculty of Medicine, University of Colombo, Colombo, Sri Lanka; 2 Department of Community Medicine, Faculty of Medicine, University of Colombo, Colombo, Sri Lanka; 3 Department of Community Medicine, Faculty of Medicine, University of Kelaniya, Kelaniya, Sri Lanka

**Keywords:** Pertussis, Sero-prevalence following childhood vaccination, Waning immunity, Adolescent booster dose

## Abstract

**Background:**

In Sri Lanka pertussis continues to circulate in the community and cases among adolescents and adults have been reported despite 95% coverage of the four dose pertussis vaccination during early childhood. Waning of immunity following natural infection or immunization may contribute to the persistent circulation. An adolescent booster dose is not included in the national immunization schedule of Sri Lanka, although this is routine practice in many countries. Therefore information on immunity to pertussis in the adolescent group is needed prior to considering vaccination schedule changes.

**Methods:**

The quantitative determination of specific Immunoglobulin G antibodies to *Bordetella pertussis* toxin was done using a commercially available validated ELISA method. The antibody values were categorized into groups according to the interpretive criteria provided by the manufacturer. The values were <55 IU/mL, negative; 55–<60 IU/mL, borderline; 60–125 IU/mL, positive; >125, strongly positive respectively. Sera of 385 asymptomatic individuals aged 4 to 24 years admitted to surgical units of Lady Ridgeway Hospital, Colombo and Colombo South Teaching Hospital were used for the study. Mann-Whitney U and Kruskal-Wallis tests were used in analysis of results and p ≤0.05 was considered as statistically significant. Details of epidemiological variables were collected using a questionnaire and correlation with significant levels of pertussis antibodies was determined.

**Results:**

Median age of the study population was 12 years with 212 (55.1%) females. The median anti PT antibody level was 3.31 IU/mL and 352 (91%) had anti PT levels ≤55 IU/mL. Median of anti PT levels were 3.18 IU/mL for 4–7 years, 1.43 IU/mL (IQR 0.336–6.27) for 8–11 years, 4.28 IU/mL (IQR 0.978–13.39) for 12–15 years, 6.14 IU/mL for 16–19 years and 4.89 IU/mL for 20–24 years and the differences were statistically significant (*p* = 0.000). Females (*p* < 0.003) and those having a sibling aged ≥12 years (*p* = 0.017) had significantly higher anti PT levels.

**Conclusions:**

The majority of the study population, especially 8 to 11 year age group had low anti PT IgG levels. The higher antibody titers in the 12–15 year age group seem to indicate infection in early adolescence. A booster dose of acellular pertussis vaccine need to be considered.

## Background

Pertussis is caused by *Bordetella pertussis*, a Gram-negative, pleomorphic coccobacillus, which is an exclusive human pathogen. Pertussis is most severe among infants, who have the highest risk of complications including pneumonia, encephalopathy and seizure leading to high morbidity and mortality [1–3]. Although newborns acquire antibodies passively from mothers, most infants are not protected during the first few months of life [4]. They are susceptible to life-threatening disease with a high incidence of deaths in the first 6 months of life [4]. Pertussis is frequently milder among older children, adolescents and adults, but they remain a significant sources of transmission of pertussis to unvaccinated young infants [5, 6].

In the world a dramatic reduction (>90%) in the incidence and mortality due to pertussis was observed following large-scale vaccination during the 1950s and 1960s [7]. However pertussis continues to circulate in the communities and estimates from WHO suggest that in 2008 about 16 million cases of pertussis occurred worldwide, 95% of which were reported in developing countries [7]. Furthermore, many countries with high vaccination coverage has reported a resurgence of pertussis recently and it includes many European countries, USA and Australia and many others [8]. Thus pertussis remains a poorly controlled vaccine preventable diseases in the world.

Although the exact causative factors are yet to be defined for the persistent circulation of pertussis, it may be due to the following reasons: waning of immunity induced by immunization, better awareness of the disease, improvement of the diagnostic methods and adaptation of *Bordetella pertussis* to survive in vaccinated populations. Whole cell pertussis vaccine (wP) which is composed of killed entire bacteria induces a broad immune response against many bacterial antigens (including PT, FHA). Estimates of the duration of immunity provided by whole-cell vaccine range from 4 to 12 years [9]. It is estimated that immunity following acellular pertussis vaccination begins to decline after 4 to 5 years [10]. These factors suggest that a booster dose may be required in the adolescent age groups irrespective of the type of vaccine received during infancy.

The recognition of the predominant role of adult as a source of transmission to infants and increased incidence of pertussis among adults suggest potential benefits in providing a booster dose to adolescents and adults [1]. The adolescent booster dose of dTap (acellular pertussis), is included in the lists of recommended immunizations in several countries including Canada, Australia, France and Germany [11].

With regard to data from the South Asian region, the Indian Academy Paediatrics has recommended a single dose of the vaccine to adolescents aged 10–12 years. However, there is no data on the coverage of acellular pertussis vaccine in adolescents and adults in India since it is being used exclusively in private health sector [12]. Even though the Global Pertussis Initiative (GPI) recommends the incorporation of adolescent booster dose of pertussis for the South Asian countries, almost none have included this in their respective national immunization programme. Lack of disease surveillance practices, difficulties in establishing the diagnosis and lack of awareness among public, health care professionals and government policy makers are identified as possible reasons for this stance. It is evident that these fundamentals need to be addressed so that pertussis prevention strategies recommended by the GPI can be implemented [13].

In Sri Lanka, combined diphtheria, tetanus and whole cell pertussis vaccines (DTwP), has been part of the state funded National Immunization Programme (NIP) since 1961. In 2009 combined pentavalent DPwT-Hepatitis B-Haemophillus Inluesnza B vaccine was introduced and is given at 2, 4, 6 months with a dose of DTwP vaccine at 18 month of age [14]. Currently, adolescent dTap vaccination is not included in the state funded National Immunization Programme.

Most developing countries use the whole cell pertussis vaccination (DTwP), due to low cost. Since there is inadequate data on waning of pertussis specific antibody levels following whole cell pertussis vaccination it would be important to determine the degree of protection after vaccination.

Therefore the study was conducted to determine the level of immunity against pertussis in an age stratified target group who has previously received childhood whole cell pertussis vaccination. It was also decided to determine any association between antibody titers and gender and exposure to smoking which have been previously recognized as factors significantly associated with occurrence of pertussis [1]. Since adolescents are well identified as a source of infection in pertussis the presence of a ≥12 year old sibling was also included as an associated factor.

## Methods

### Research plan

A descriptive cross sectional study was carried out during the period January to April 2014. A study unit was defined as a previously healthy individual aged 4–24 years admitted for surgical or trauma care at the Colombo South Teaching Hospital or Lady Ridgeway Hospital or Children who had received the DTwP immunization according to the National Immunization Proramme (NIP) of Sri Lanka. Serum samples of 385 consequent admissions aged between 4 and 24 years old, admitted to surgical and trauma units of Colombo South Teaching Hospital (CSTH) and Lady Ridgeway Hospital (LRH) Sri Lanka were used for the study. Subjects who had received DTwP vaccine at 2, 4, 6 and 18 months as per NIP of Sri Lanka were included. Immunization status was ascertained by interviewing the subject/parents followed by checking the evidence of immunization in the Child Health Development Record. Individuals who had symptoms or signs of systemic illness at the time of data collection, a complaint of cough for more than 2 weeks in the previous 3 months, or who had ever received a booster dose of dTap (acellular pertussis) vaccine or diagnosed with immunocompromising conditions or received immunosuppressive agents were excluded from the study.

Sample size was calculated as 384 to detect an unknown prevalence of immunity against Bordetella pertussis (estimated as 50%) with an α error of 5% and a precision level of 5%. Patients conforming to the eligibility criteria were consecutively selected from all the surgical units in the two hospitals until the required sample size was achieved.

### Data collection

An interviewer administered questionnaire, a face to face interview with the patient or guardian and the Child Health Development Record were used to determine immunization status and socio-demographic characteristics of the study group. Active smoking was assessed by asking the participants about current smoking in any amount and frequency. Passive smoking was identified as a participant living in the same household with a current smoker who smokes inside the premises, This was followed by collection of the residual serum from the blood samples sent for routine investigations (blood urea and serum electrolytes) at the chemical pathology laboratory and for blood grouping and direct test (BGDT) at the blood bank prior to surgery. Blood samples were coded using the same reference number used in the questionnaire.

### Measurement of IgG levels by Enzyme Linked Immuno-Sorbent Assay (ELISA)

The IgG levels in IU/mL was measured using the commercially available validated ELISA kit (Sero Pertussis Toxin IgG, Catalog No.1231-01D, Savyon Diagnostics Ltd, Ashdod, ISRAEL) for the quantitative determination of specific IgG antibodies to Pertussis toxin in human serum.

The wells of solid phase ELISA has been coated with purified pertussis toxin (PT) antigen. The serum samples were added to the wells and specific antibodies present in serum against pertussis antigen (anti-PT antibodies) were made to bind to the immobilized antigen. The unbound nonspecific antibodies were removed by washing. In the next step anti-human IgG conjugated to horseradish peroxidase (HRP) was added which is expected to bind to the antigen-antibody complex. The unbound conjugate was removed by washing and the TMB- substrate was added which was hydrolyzed by the peroxidase, yielding a yellow color. Subsequently the stop solution was added to halt the reaction and the absorbance was measured using the spectrophotometer at a wavelength of 450/620 nm. It was inferred that the absorbance is proportional to the level of IgG-specific antibodies that are bound to the antigens. The test results were calculated using the standard curve and interpreted according to the manufacturer’s instructions. ELISA was validated using the specific test validation conditions specified by the manufacturer and the standard curve was plotted using the optical density (OD) values of calibrated standards with known titers.

SeroPertussis Toxin IgG; Savyon® Diagnostics Ltd., Ashdod, Israel ELISA kit comprising purified PT as the antigen, designed to detect anti-PT IgG antibodies has been validated by comparing with in-house reference ELISA in terms of specificity, and sensitivity by Dinu et al. [15]. The reference in-house ELISA with purified PT and NIBSC reference sera was used as described in Guiso et al. [22] to assay anti-pertussis toxin IgG antibodies. The estimated sensitivity and specificity of this test for anti-PT IgG antibodies, with reference to the results of the in-house ELISA, were 88 and 100%, respectively. Sixty five serum samples from patients suspected to have pertussis were tested by three different investigators using two batches of the test kit. The Intra assay agreement was good and the differences never exceeding 17%. Thus, the authors have concluded that this kit is comparable to other validated ELISAs used for testing anti-PT IgG antibodies [15].

The anti PT antibody titers in IU/mL were categorized into groups according to the interpretive criteria provided by the manufacturer [16]. They were <55 IU/mL–negative, 55–<60 IU/mL–borderline, 60–125 IU/mL–positive and >125 IU/mL–strongly positive.

### Statistical analysis

The data were analyzed using SPSS17 and a *p* value ≤0.05 was considered as statistically significant. The subjects were divided into five groups of equal width according to age ranges, 4 to 7 years, 8 to 11 years, 12 to 15 years, 16 to 19 years, and 20 to 24 years. The mean, medium, maximum and minimum anti PT values were calculated for each age group.

The Mann-Whitney *U* Test was used in determining the association of antibody titers with gender, exposure to smoking (either active or passive) and having ≥12 year old sibling.

## Results

Among the sample of 385 individuals, 55.06% (212) were females. The mean age of the sample was 13.20 years, the median 12.00 years (IQR 8–19), and range 4 to 24 years.

### Anti PT antibody titers in the whole study population

**Fig. 1:**
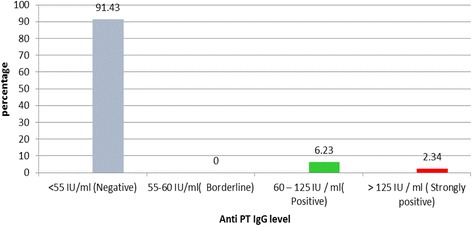
Anti PT IgG antibody levels in the study population

The anti PT antibody levels of the study sample ranged from 0.0153 IU/mL to 149.76 IU/mL and the median value was 3.3108 IU/mL (IQR 0.73–15.12). Three hundred fifty-two out of 385 (91.43%) had anti-PT levels ≤55 IU/mL, indicating non-significant levels of antibody in a substantial proportion. 6.23% (24 out of 385) of study group had anti PT levels between 60 and 125 IU/mL (positive) while 2.34% (9 out of 385) had anti PT levels exceeding 125 IU/mL (strongly positive). None of the participants in the study group belonged to the borderline category (55 to 60 IU/mL) (Fig. 1).

### Anti PT antibody titers and age stratified cohorts

**Fig. 2:**
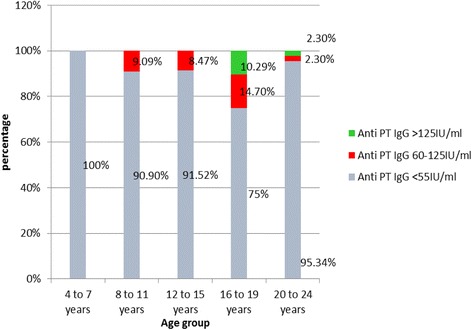
Age stratified anti PT IgG antibody levels in the study population

For further analysis of results the study sample was age stratified into five groups and age group related median anti PT antibody titers are shown in Table 1. The median anti PT value of the 4 to 7 year group was 3.18 IU/mL (IQR 0.591–8.00). The lowest median value of anti PT antibody level, 1.43 IU/mL (IQR 0.336–6.27) was seen in the 8 to 11 year age group. The median anti-PT value then increased gradually, to 4.28 IU/mL (IQR 0.978–13.39) in the 12 to 15 year group, reaching the highest value of 6.14 IU/mL (1.44–63.35) in the 16 to 19 year age group. The values in the group aged 20 to 24 years showed a decline in the titer to 4.89 IU/mL (IQR 1.11–16.78) as depicted in Table 1 and Fig. 2. Thus the highest median titre was detected in the 16–19 year age cohort. Spearman Correlation Coefficient test confirmed that there was a statistically significant correlation between anti PT IgG value and age (*P* = 0.0121).

**Table 1:** Age distribution and the median anti PT IgG levels of the study population

Age group	Number (%)	Median Anti-PT IgG IU/ml	IQR
A (4–7 years)	95 (24.68)	3.18	0.59–8.00
B (8–11years)	77 (20.0)	1.43	0.34–6.27
C (12–15 years)	59 (15.32)	4.28	0.98–13.39
D (16–19 years)	68 (17.66)	6.14	1.44–63.25
E (20–24 years)	86 (22.34)	4.89	1.11–16.78

### Anti PT antibody titers of the age stratified study populations

**Fig. 3:**
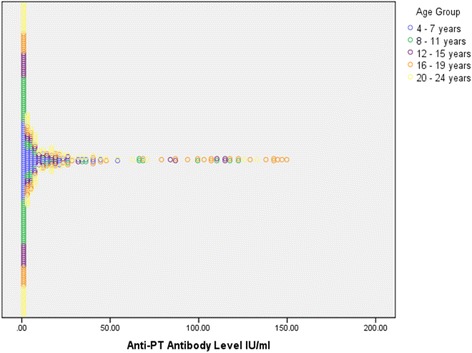
Distribution of Anti-PT antibody levels in the five age groups

Table 2 provides information on antibody titre distribution in different age cohorts. The highest number of children with significant titers of antibodies were found in the 16–19 year age cohort. Figure 3 shows the distribution of actual values of ant-PT antibody levels in the five age groups.

**Table 2:** The anti-PT IgG titre distribution in different age groups

Age group (years)	<55 IU/mL Number (%)	60–125 IU/mL Number (%)	>125 IU/mL Number (%)
4 to 7 (*n* = 95)	100	0	0
8 to 11 (*n* = 77)	90.9	9.09	0
12 to 15 (*n* = 59)	91.52	8.47	0
16 to 19 (*n* = 68)	75	14.7	10.29
20 to 24 (*n* = 86)	95.34	2.3	2.3

### Association between gender and the median anti PT level

55.06% of the population were females while 44.94% were males. The median anti-PT IgG levels were substantially higher in females (4.6367 IU/ml) (IQR 4.6367) than in male (2.5013 IU/mL) (IQR 2.501) subjects. According to the Mann-Whitney test females (*p* < 0.003) had significantly higher anti PT levels (Table 3).

**Table 3:** Factors associated with anti-PT levels

Associated factors		No.	Median anti-PT level (IU/ml)	P* value
Gender	Girl	212	4.6367	<0.003
	Boy	173	2.5013	
Having a sibling aged more than >12 years	Yes	176	4.840	0.017
	No	209	2.7654	
Exposure to either active or passive smoking	Yes	27	2.50	0.882
	No	358		

*p value was calculated with Mann-Whitney test

### Association between median anti PT level and having a sibling ≥12 years

Among the study participants, 54.29% of the subjects had a sibling ≥12 years. The median anti-PT IgG level of subjects having siblings ≥12 years old was 4.830 IU/mL (IQR 1.0461–17.4588), and was higher than the group that did not have a sibling ≥12 years (2.765400 IU/mL) (IQR 0.6264–11.5587). This difference was statistically significant (*p* = 0.017) with the Mann-Whitney test (Table 3).

### Association between median anti PT level and exposure to either active or passive smoking

Only 7.01% of the study group had been exposed to either active or passive smoking while 92.99% had not been exposed to either. The median anti PT IgG value of subjects who were not exposed to smoking were higher (3.320 IU/mL) (IQR 0.7105–15.290) than those who were exposed to smoking (2.50 IU/mL) (IQR 0.8148–17.011) (Table 3). However this difference was not shown to be significant by the Mann-Whitney test (*p* = 0.882).

## Discussion

The incidence of pertussis has shown an increase during the last decade, even in countries with high vaccine coverage [17, 18]. In the recent past clinical cases of pertussis manifested as outbreaks among adolescents and adults in certain countries including the Netherlands, USA and several other European countries [17–19]. According to the WHO, 42,826 cases were reported in South East Asia during 2010 which increased to 45,847 during 2012 [20]. This changing global trend is explained as, due to increased awareness, improved diagnostic methods, and waning of the vaccine induced immunity [10]. In Sri Lanka, according to the hospital statistics over 1000 cases of pertussis per year were reported until the late 1970, and a gradual decline in the number of cases was observed since 1990. However, a total of 24 clinically suspected pertussis cases were reported all over the country during 2010 [14]. It was followed by 23 cases in 2011 and 61 cases in 2012 showing an increasing trend [20].

The protective immunity of pertussis may be mediated by both humoral and cell mediated immunity. However, the anti-pertussis antibodies are still considered as the best surrogate marker of immunity and determination of which would be important to detect waning immunity against the disease [1]. The determination of age associated antibody levels against *B pertussis* is important in deciding the target age group for booster vaccination as well as for the study of disease epidemiology [21].

The median anti PT antibody level was 3.31 IU/mL and 352 (91%) had anti PT levels ≤55 IU/mL which was a very low level of seropositivity. Median of anti PT levels were 3.18 IU/mL for 4–7 years, 1.43 IU/mL (IQR 0.336–6.27) for 8–11 years, 4.28 IU/mL (IQR 0.978–13.39) for 76 12–15 years, 6.14 IU/mL for 16–19 years and 4.89 IU/mL for 20–24 years and the differences were statistically significant (*p* = 0.000). Females (*p* < 0.003) and those having a sibling aged ≥12 years (*p* = 0.017) had significantly higher anti PT levels. According to the results of this study the majority of the study population showed low seropositivity. This infers that a significant proportion of the study population is probably having a risk of acquiring infection as well as contributing to the reservoir group responsible for transmission of infection. In a study which compared the serological response to whole cell pertussis lysate and PT, a lower prevalence of antibodies to PT was shown [1]. The study suggested that an assay that uses the whole cell lysate which includes both PT and FHA is far more sensitive than the PT antigen alone in determining antibody titers [1]. However PT is recognized to be the only *B.pertussis* specific antigen and for routine diagnosis the measurement of anti-PT antibodies alone has been recommended [22]. Therefore the use of anti PT antibody levels to determine immunity against pertussis is acceptable.

The majority of the study population as a whole showed very low levels of sero positivity (92%). With regard to age stratified antibody titers, the 16 to 19 age group showed the highest levels of significant seropositivity amounting to a proportion of 25% of the specific age cohort. Since all subjects in the study group had not received an adolescent booster dose, this high IgG levels may indicate natural exposure to the pathogen. The median anti PT IgG level was lowest in the 8 to 11 year age group. Several other studies done to determine the sero-prevalence of pertussis has shown similar findings [23]. A study done in the Czech Republic to determine levels of the anti-PT antibodies after childhood vaccination showed that seropositivity in children declines rapidly after the last dose of vaccination and the lowest level of anti PT antibodies were observed among preschool age group [24]. According to a study conducted in Turkey, where the final dose of whole cell pertussis vaccine is given at the age of 18 months [23], the lowest geometric titer for the anti-pertussis antibodies were observed in the 4 to 6 years age group and 51.7% of this group were negative for pertussis antibodies [23]. Similarly a study conducted among Iranian medical students has reported a sero positivity of 47.6%. A considerable proportion of this study population with a positive history of childhood vaccination for pertussis was also not serologically immune to pertussis [25]. A sero-epidemiological study of pertussis conducted in China using a wider age range of 0–95 years also reports very low sero-positivity rate all the age groups. Highest mean antibody levels (18.44 IU/ml) and sero-positivity (11.43%) was reported in 13–19 age group which suggests peak incidence of B. pertussis infection in this age group. This finding is very much similar to the findings of the present study [26].

In the present study, the median anti PT IgG value gradually increased from 12 to 15 year age group and the highest levels were observed among the 16 to 19 year age group. This type of increasing sero-prevalence with age has been observed with good immunization coverage during childhood [27]. A study conducted in Turkey, where the final dose of whole cell pertussis vaccine is given at the age of 18 months, the pertussis antibodies were highest at 51.4 U/mL in the 13 to 18 years age group similar to findings from this study [23]. In contrast, a study done in Nashville, where the last vaccine dose is given at the age of 4 to 6 years, two peaks of antibody titres were noted, one at the age range of 4 to 6 years while the other at 13 to 17 years [28]. These findings suggest that vaccination schedules practiced in different countries appears to affect the seropositivity patterns observed in different age groups. Further, there may be other factors such as vaccine immunogenicity and genetic factors which determine the maintenance of antibody levels.

The anti PT IgG levels are considerably higher in the female population when compared to the male population in this research study. This disease is considered more common among females globally and whether this is due to higher immunogenicity of the female population or higher exposure to disease is yet to be defined [1].

The subjects who had a sibling ≥12 years had higher antibody levels when compared with those who didn’t. The adolescents may act as reservoirs for the maintenance of pertussis and are considered as important sources for the continued circulation of pertussis [27]. In this study the exposure to active or passive smoking was not associated with the titers of immunoglobulin although other research studies have shown such an association [1]. The small number of subjects with this risk factor may have affected the results of this study.

Findings of this study needs to be interpreted in the light of certain limitations. This was conducted in a hospital based sample and therefore will not be exactly representative of the general population of that age group. However, the study population was selected from previously healthy individuals who were admitted to the surgical and trauma units for elective minor surgeries and trauma care, and all those with signs of systemic illness were excluded. Whilst this population does not exactly represent a community-based study population, due to the selection of previously healthy participants from surgical units helped to minimize any selection bias. The fact that these participants sought surgical care in these hospitals is unlikely to be related to their pertussis immunity status and because of that this sample is likely to reasonably represent the immunity status for pertussis in the general population. Furthermore, selecting a hospital based sample from whom blood is routinely drawn for investigations has helped to avoid any selection bias that may occur by selecting volunteers who would agree to participate in the study due to concerns regarding their immunity status. Sampling by selection of patients consecutively from all the selected units could not have introduced any additional bias to the study. The study kit used for the determination of antibody levels was a previously validated kit [15]. However, if two methods of antibody detection were used it would have contributed to increase the validity of the data, compared to using a single test kit.

## Conclusions

The pertussis infection in early adolescence may provide the reservoir for transmission of the infection to infants and young children who suffer from a more complicated course of disease.

The evidence of waning immunity after vaccination and the evidence for recurrent infection with pertussis in this study highlights the potential benefit of the immunization of adolescents.

The vaccination of adolescents with safe and highly immunogenic acellular vaccine is currently practiced in several countries globally. Since Sri Lanka is having an admirable compliance to immunization within the population, it appears logical to control infections in infants by reducing the reservoir population. A booster dose of the acellular pertussis vaccine may help in reducing infection among adolescents due to waning immunity. This is expected to lower pertussis infection and related complications among infants and young children.

A booster dose of acellular pertussis vaccine may be indicated to 8 to 11 year age groups as per serological profiles observed in this study. However, additional information on the incidence of infection in the adolescent group accompanied by a large population based study may be required prior to implementation of the above recommendation.

## Abbreviations

BGDT: Blood grouping and direct test
CSTH: Colombo South Teaching Hospital
dTaP: Combined reduced diphtheria, tetanus and acellular pertussis vaccine
DTwP: Combined diphtheria, tetanus and whole cell pertussis vaccine
ELISA: Enzyme Linked Immuno-Sorbent Assay
FHA: Filamentous Haemagglutinin antigen
GPI: Global Pertussis Initiative
HRP: Horseradish peroxidase
IQR: Inter-quartile range
LRH: Lady Ridgeway Hospital
NIBSC: National Institute for Biological standards and Control
NIP: National Immunization Programme
OD: Optical density
PT: Pertussis Toxin
WHO: World Health Organization
wP: Whole-cell Pertussis Vaccine

## References

[1] Arav-Boger R, Ashkenazi S, Gdalevich M, Cohen D, Danon YL (2000). Seroprevalence of Pertussis Antibodies among Adolescents. IMAJ.

[2] Greenberg DP, von Konig CW, Heininger U (2005). Health Burden of Pertussis in Infants and Children. Pediatr Infect Dis J.

[3] Heininger U, Klich K, Stehr K, Cherry JD. Clinical Findings in *Bordetella pertussis* Infections: Results of a Prospective Multicenter Surveillance Study. Pediatrics. 1997. http://www.pediatrics.org/cgi/content/full/100/6/e10. Accessed 20 May 2014. 10.1542/peds.100.6.e10 9382911

[4] Nooitgedagt JE, de Greeff SC, Elvers BH, de Melker HE, Notermans DW, Huisseling HV, Versteegh FGA (2009). Seroprevalence of *Bordetella pertussis* Infection during Pregnancy Measured by IgG Antibodies against Pertussis toxin. Clin Infect Dis.

[5] David ST, Hemsley C, Pasquali PE, Larke B, Buxton JA, Lior LY (2006). Enhanced Surveillance for Adverse Events Following ImmunizationTwo Years of dTap Catch-Up Among High School Students in Yukon, Canada (2004, 2005). Can J Public Health.

[6] Wendelboe AM, Hudgens MG, Poole C, Rie AV (2007). Estimating the role of casual contact from the community in transmission of *Bordetella pertussis* to young infants. Emerg Themes Epidemiol.

[7] World Health Organisation. WHO poistion paper: Pertussis vaccines. 2010. 85(40):385-400. 20939150

[8] Fisman DN, Tang P, Hauck T, Richardson S, Drews SJ, Low DE, Jamieson F (2011). Pertussis resurgence in Toronto, Canada: a population-based study including test-incidence feedback modeling. BMC Public Health.

[9] Wendelboe AM, Rie AV, Salmaso S, Englund JE (2005). Duration of Immunity Against Pertussis After Natural Infection or Vaccination. Pediatr Infect Dis J.

[10] Forsyth KD, Campins-Marti M, Caro J, Cherry JD, Greenberg D, Guiso N, Heininger U, Schellekens J, Tan T, von Konig CH, Plotkin S (2004). New Pertussis Vaccination Strategies beyond Infancy, Recommendations by the Global Pertussis Initiative. Clin Infect Dis.

[11] Tan T, Trindade E, Skowronski D (2005). Epidemiology of Pertussis. Pediatr Infect Dis J.

[12] Vashishtha VM, Bansal CP, Gupta SG (2013). Pertussis Vaccines: Position Paper of Indian Academy of Pediatrics (IAP). Indian Pediatr.

[13] Forsyth K, von König CHW, Tan T, Plotkin S (2012). Pertussis control in the Asia-Pacific region: a report from the Global Pertussis Initiative. Southeast Asian J Trop Med Public Health.

[14] Epidemiology Unit (2012). Immunization Handbook.

[15] Dinu S (2014). Whooping cough in South-East Romania: a 1-year study. Diagn Microbiol Infect Dis.

[16] SeroPertussis Toxin IgG, Enzyme Linked Immunosorbent Assay (ELISA) for the quantitative determination of specific IgG antibodies to Bordetella Pertussis Toxin in human serum. Israel: Instruction Manual, Savyon Diagnostics Ltd. www.savyondiagnostics.com.

[17] Hellenbrand W, Beier D, Jensen E, Littmann M, Meyer C, Oppermann H, von König CW, Reiter S (2009). The epidemiology of pertussis in Germany: past and present. Bio Med Cent Infect Dis.

[18] de Melker HE, Schellekens JFP, Neppelenbroek SE, Mooi FR, Rümke HC, Conyn-van Spaendonck MAE (2000). Re-emergence of Pertussis in the Highly Vaccinated Population of the Netherlands: Observations on Surveillance Data. Emerg Infect Dis.

[19] Guris D, Strebel PM, Bardenheier B, Brennan M, Tachdjian R, Finch E, Wharton M, Livengood JR (1999). Changing Epidemiology of Pertussis in the United States: Increasing Reported Incidence Among Adolescents and Adults, 1990–1996. Clin Infect Dis.

[20] World Health Organisation. Expand Programme of Imunisation Fact Sheet. South East Asian Regional Office. 2012.www.searo.who.int/entity/immunisation/data/EPI_FactSheet-Regional_2012.pdf.

[21] Cagney M, Macintyre CR, McIntyre P, Puech M, Giammanco A (2006). The seroepidemiology of pertussis in Australia during an epidemic period. Epidemiol Infect.

[22] Guiso N, Berbers G, Fry NK, He Q, Riffelmann M, von König CHW (2011). What to do and what not to do in serological diagnosis of pertussis: recommendations from EU reference laboratories. Eur J Clin Microbiol Infect Dis.

[23] Cevik M, Beyazova U, Aral AL, Camurdan AD, Ozkan S, Sahin F, Aybay C (2008). Seroprevalence of IgG antibodies against Bordetella pertussis in healthy individuals aged 4–24 years in Turkey. Clin Microbiol Infect.

[24] Durpektova M, Hrstkova H (2008). Is the level of IgG antibodies aginst Pertussis toxin sufficient in vaccinated child population?. Scr Med.

[25] Hashemi SH (2009). Seroprevalence of Immunoglobulin G antibodies against pertussis toxin among asymptomatic medical students in the west of Iran: a cross sectional study. BMC Infect Dis.

[26] Zhang Q (2012). The seroepidemiology of Immunoglobulin G antibodies against pertussis toxin in China: a cross sectional study. BMC Infect Dis.

[27] Strebel P, Nordin J, Edwards K, Hunt J, Besser J, Burns S, Amundson G, Baughman A, Wattigney W (2001). Population-Based Incidence of Pertussis among Adolescents and Adults Minnesota, 1995–1996. J Infect Dis.

[28] Cattaneo LA, Reed GW, Haase DH, Wills MJ, Edwards KM (1996). The Seroepidemiology of *Bordetella pertussis* Infections: A Study of Persons Ages 1–65 Years. J Infect Dis.

